# Effects of heat and personal protective equipment on thermal strain in healthcare workers: part B—application of wearable sensors to observe heat strain among healthcare workers under controlled conditions

**DOI:** 10.1007/s00420-023-02022-2

**Published:** 2023-11-10

**Authors:** Razan Wibowo, Viet Do, Caroline Quartucci, Daniela Koller, Hein A. M. Daanen, Dennis Nowak, Stephan Bose-O’Reilly, Stefan Rakete

**Affiliations:** 1 Institute and Clinic for Occupational, Social and Environmental Medicine, University Hospital, LMU Munich, 80336 Munich, Germany; 2 Institute for Occupational Safety and Environmental Health Protection, Bavarian Health and Food Safety Authority, 80538 Munich, Germany; 3 Institute for Medical Information Processing, Biometry and Epidemiology, LMU Munich, 81377 Munich, Germany; 4 Faculty of Behavioural and Movement Sciences, Vrije Universiteit Amsterdam, Amsterdam, The Netherlands; 5 Institute of Public Health, Medical Decision Making and Health Technology Assessment, Department of Public Health, Health Services Research and Health Technology Assessment, UMIT-University for Health Sciences, Medical Informatics and Technology, Hall in Tirol, Austria

**Keywords:** Climate change, Healthcare worker, Heat stress, Personal protective equipment, Wearables, Physiological effects

## Abstract

**Purpose:**

As climate change accelerates, healthcare workers (HCW) are expected to be more frequently exposed to heat at work. Heat stress can be exacerbated by physical activity and unfavorable working requirements, such as wearing personal protective equipment (PPE). Thus, understanding its potential negative effects on HCW´s health and working performance is becoming crucial. Using wearable sensors, this study investigated the physiological effects of heat stress due to HCW-related activities.

**Methods:**

Eighteen participants performed four experimental sessions in a controlled climatic environment following a standardized protocol. The conditions were (a) 22 °C, (b) 22 °C and PPE, (c) 27 °C and (d) 27 °C and PPE. An ear sensor (body temperature, heart rate) and a skin sensor (skin temperature) were used to record the participants´ physiological parameters.

**Results:**

Heat and PPE had a significant effect on the measured physiological parameters. When wearing PPE, the median participants’ body temperature was 0.1 °C higher compared to not wearing PPE. At 27 °C, the median body temperature was 0.5 °C higher than at 22 °C. For median skin temperature, wearing PPE resulted in a 0.4 °C increase and higher temperatures in a 1.0 °C increase. An increase in median heart rate was also observed for PPE (+ 2/min) and heat (+ 3/min).

**Conclusion:**

Long-term health and productivity risks can be further aggravated by the predicted temperature rise due to climate change. Further physiological studies with a well-designed intervention are needed to strengthen the evidence for developing comprehensive policies to protect workers in the healthcare sector.

**Supplementary Information:**

The online version contains supplementary material available at 10.1007/s00420-023-02022-2.

## Introduction

Climate change is one of the major challenges of our time (Trenberth 2018; UNEP 2021). In Germany, the average temperature has increased by more than 1.5 °C since 1881 and extreme weather events such as hot summer days (> 30 °C) and heat waves (multiple consecutive days with > 30 °C) will occur more frequently (Papalexiou et al. 2018; IPCC 2019; van Rüth et al. 2019).Climate change affects many occupations in terms of heat stress, e.g. road and agricultural workers. However, indoor temperatures may also reach subjectively uncomfortable but also health-relevant levels as air-conditioned workplaces are not common in Germany (Lenzer et al. 2020). In Germany, the German Society for Occupational and Environmental Medicine (Deutsche Gesellschaft für Arbeitsmedizin und Umweltmedizin e. V. (DGAUM)) defines “working under heat stress” as climatic stress in the workplace caused by an extreme increase in indoor/outdoor temperature due to heat (DGAUM 2012). Increased indoor temperatures due to hot weather are known to cause heat strain (Simister and Cooper 2005). Amongst others, healthcare workers (HCW) such as nurses have an increased risk of heat strain due to comparatively high job-related physical activity (Schoierer et al. 2019). This may be further aggravated by the use of personal protective equipment (PPE) (Dorman and Havenith 2009).

Heat strain is the overall physiological response resulting from heat stress. If the core body temperature exceeds normal levels (36.8–37.5 °C) and the thermoregulatory system fails to compensate the heat stress, the risk of heat strain increases (Mazlomi et al. 2017; Ebi et al. 2018). A core body temperature above 38 °C can lead to fatigue, headache, dizziness, loss of appetite and rapid breathing (Gostimirovic et al. 2020). Heat stress triggers the production of stress hormones such as adrenaline, noradrenaline, and cortisol (McMorris et al. 2006). This may explain in part the physiological responses of heat stress such as an increase of core and peripheral temperature, heart rate, and sweating (Mazlomi et al. 2017). Multiple studies have shown that long-term exposure to heat reduces the work capacity and increase mortality rates within the general population (Rowell 1974; Arbury et al. 2014; Steul et al. 2018; an der Heiden et al. 2019; Bisolli et al. 2019; Vicedo-Cabrera et al. 2021).

Although body temperature and other physiological parameters can help to detect symptoms of heat strain, their monitoring in occupational settings is still challenging. As wearable devices are becoming more advanced, the online monitoring of selected health parameters may help to better understand and monitor changes due to increased temperature (Notley et al. 2018). In general, wearable devices consist of three main components: (1) the hardware measuring physiological and/or activity data, (2) the communication hardware and software to relay data to a (remote) processing unit and (3) the data analysis techniques to extract clinically relevant information from the obtained data (Patel et al. 2012). Their current capabilities include physiological, biochemical and motion sensing (Teng et al. 2008; Bonato 2010). However, wearable devices to assess physiological parameters related to heat strain due to occupational heat stress among HCW, have not been used thus far.

Therefore, the goal of this study was the feasibility assessment of wearable devices for the monitoring of physiological parameters of HCW during controlled heat stress conditions. Furthermore, the individual effects of high temperatures and PPE on these parameters were investigated.

## Materials and methods

### Study design and experimental setup

This study was designed as a crossover trial, which took place between October 2021 and March 2022. The experiments took place in climate chamber (5 × 3 × 2.2 m (L/W/H)) with temperature and humidity control. The interior included a table with a chair, a treadmill, a 170 patient bed and patient dummy (CLA1®, 21 kg, Coburger Lehrmittelanstalt, Coburg, Germany). A photo of the climate chamber setting can be found in the supplemental information (Fig. S1).

Four independent experiments were performed by each the participants: (1) at 22.0 °C without PPE (NN), at 22 °C with PPE (NP), (3) at 27 °C without PPE (WN) and (4) at 27 °C with PPE (WP). The selection of the higher temperature was based on the guidelines of the German Federal Ministry of Labour and Social Affairs, recommending that the ambient temperature in workspaces should not exceed 26 °C (BMAS 2022). However, we chose 27 °C in order to achieve a sufficient temperature difference to the reference temperature of 22 °C. The relative humidity was set to 40% for all four scenarios to limit the number of experimental variables. Participant wore a standard hospital gown and additional PPE (disposable plastic gown, FFP2 face mask, face shield and gloves) if applicable. To alleviate a habituation or training effect, the setting´s sequence was randomized. During the experiment participants performed HCW-related activities within a given time. These activities include, amongst others, walking up to 5.8 km/h on a treadmill, mobilization and washing of a dummy and the simulation of administrative tasks. The full protocol can be found in the supplementary information. Except for temperature and PPE, all other conditions were identical. One experiment lasted 3.5 h. Each participant conducted all experiments at the same time of the day (either mornings or afternoons) and the interval between two individual experiments was at least seven but maximum ten days. These prerequisites aimed to minimize interfering effects of circadian rhythms and differences in the participants´ physiological condition (Goel et al. 2013).

### Participants

Inclusion of eligible participants was based on the medical history, by considering the following criteria: (1) age 18–60 years; (2) medical background or experience as HCW, since they had to perform several HCW-related activities; (3) no sensitivity against heat (e.g., dizziness, redness on the skin); (4) no obesity (BMI < 30); and (5) no severe chronic diseases. Prior to each experiment, participants were asked about their general state of health. Furthermore, heart rate, blood pressure and body temperature (forehead) were measured to exclude illnesses that may interfere with the study.

### Monitoring of the environmental conditions

A *QUESTemp 34* Heat Stress Monitor^®^ (Quest Technologies, Wisconsin, USA) was utilized for assessing the heat stress by the environmental conditions in the climate chamber. The instrument was placed on a table at a height of approximately one meter. It was also ensured that this device was placed away from any barriers that might block radiant heat or flow. The participants were also requested not to move too close to this instrument in order to minimize variations in temperature and radiant heat. The sampling interval was one minute.

### Monitoring of physiological parameters

**Fig. 1:**
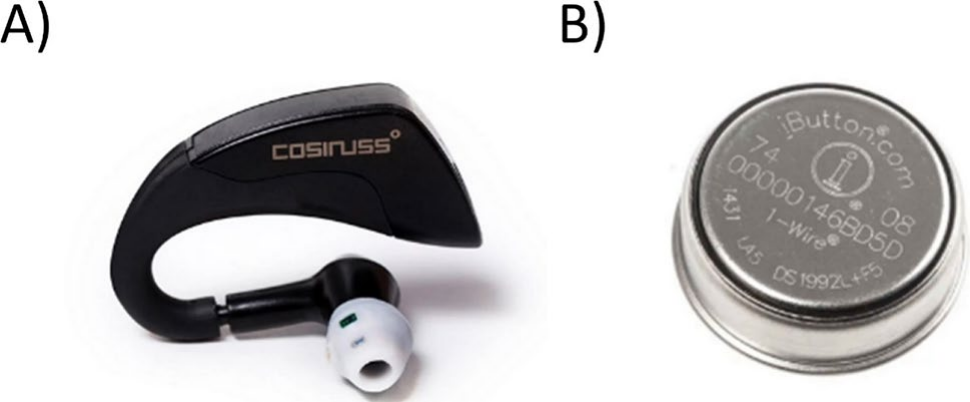
Wearable sensors used during the trials. *Cosinuss° Two* in-ear sensor® (**A**) to monitor body temperature and heart rate and *Thermochron iButton*® (**B**) to record peripheral temperatures

A c*osinuss° Two*^®^ (Cosinuss GmbH, Munich, Germany) in-ear sensor was utilized to monitor participants´ in-ear temperature (IET) and heart rate (HR) during the experiments (Fig. 1). For each participant, the appropriate sensor size (small or medium) was selected during the anamnesis. The sampling interval was one second. After 14 min of recording, the data were sent to the cosinuss° Health cloud server via a Gateway within one minute. This cycle was repeated during the whole experiment.

Moreover, skin temperatures were monitored using *Thermochron iButton*^®^ temperature loggers (CK electronic GmbH, Cologne, Germany) (Fig. 1). The sensors were placed at five different central and peripheral locations (left/right infraclavicular, belly and left/right midthigh). The sampling interval was one minute. Finally, to evaluate the participants´ clinical state, their weight, forehead temperature, blood pressure, and heart rate were measured before and after each experiment using a digital body scale, an infrared thermometer and a medical blood pressure cuff, respectively.

### Data handling and statistical analysis

Prior to the analysis, all data were pre-processed. In detail, sections outside the trials, including those with apparent sensor malfunctions, were removed and the remaining measured data were used for the analysis. Furthermore, only physiological data measured under the dry bulb indoor temperature between 20 and 24 °C for normal conditions and between 25 and 29 °C for warm conditions were included in the analysis. For in-ear temperature and heart rate, one-minute medians were calculated to match the interval of the skin sensors. Additionally, only heart rate results with corresponding signal quality index above 50 were used. The signal quality index is an algorithm quantitatively assesses the functional near-infrared spectroscopy signal quality on a numerical scale from 0 (very low quality) to 100 (very high quality). Moreover, longitudinal data from the five skin temperature measurements were corrected using an external calibration. For the calculation of the mean skin temperature (MST) for one experiment, the results of all five sensors were averaged.

Each physiological parameter was tested for normality using the Kolmogorov–Smirnov test. The analysis of variance (ANOVA) test was utilized to compare the measured data. All parameters were found to be non-normally distributed. Therefore, the Wilcoxon rank-sum test was considered. Since this method is not robust against systematic interindividual variability, a linear mixed-effects model analysis was used to properly consider the interindividual differences. The mixed model approach was broadly used in previous accelerometer studies (Van Dongen et al. 2004; Haapalainen et al. 2008; Pfeiffer et al. 2009; Bolton et al. 2021). In particular, we were interested in making conclusions about how the trial settings over time (fixed effects) impact the measured physiological parameters by controlling the individual differences (random effects). Alpha (α) level at 0.05 was set for all statistical tests. All p-values were two-tailed. The data cleaning process and statistical analysis were performed using R statistical software (version 4.1.2.^®^).

## Results

### Participants´ clinical characteristics

Eighteen participants (n = 11 females, n = 7 males) completed all four experimental sessions (with a mean interval of 8 days). The majority of them are actively working as HCW (nurses). Their mean age was 35.2 ± 10.4 years old (22–57). Pre-post-increases were observed in participants´ weight and forehead temperature. Table 1 presents all participants’ clinical characteristics from the medical history.

**Table 1:** Participant’s characteristics at the time of the preliminary examination (*n* = 18)

Parameter	Mean ± SD
Age in years	35.2 ± 10.4
Height in m	1.71 ± 0.09
Weight in kg	70.3 ± 15.8
BMI in kg/m^2^	23.9 ± 4.2
Blood pressure (sys/dia) in mmHg	137/87 ± 19/11
Heart rate in beats/min	73 ± 11

### Measured data from the used instruments

**Fig. 2:**
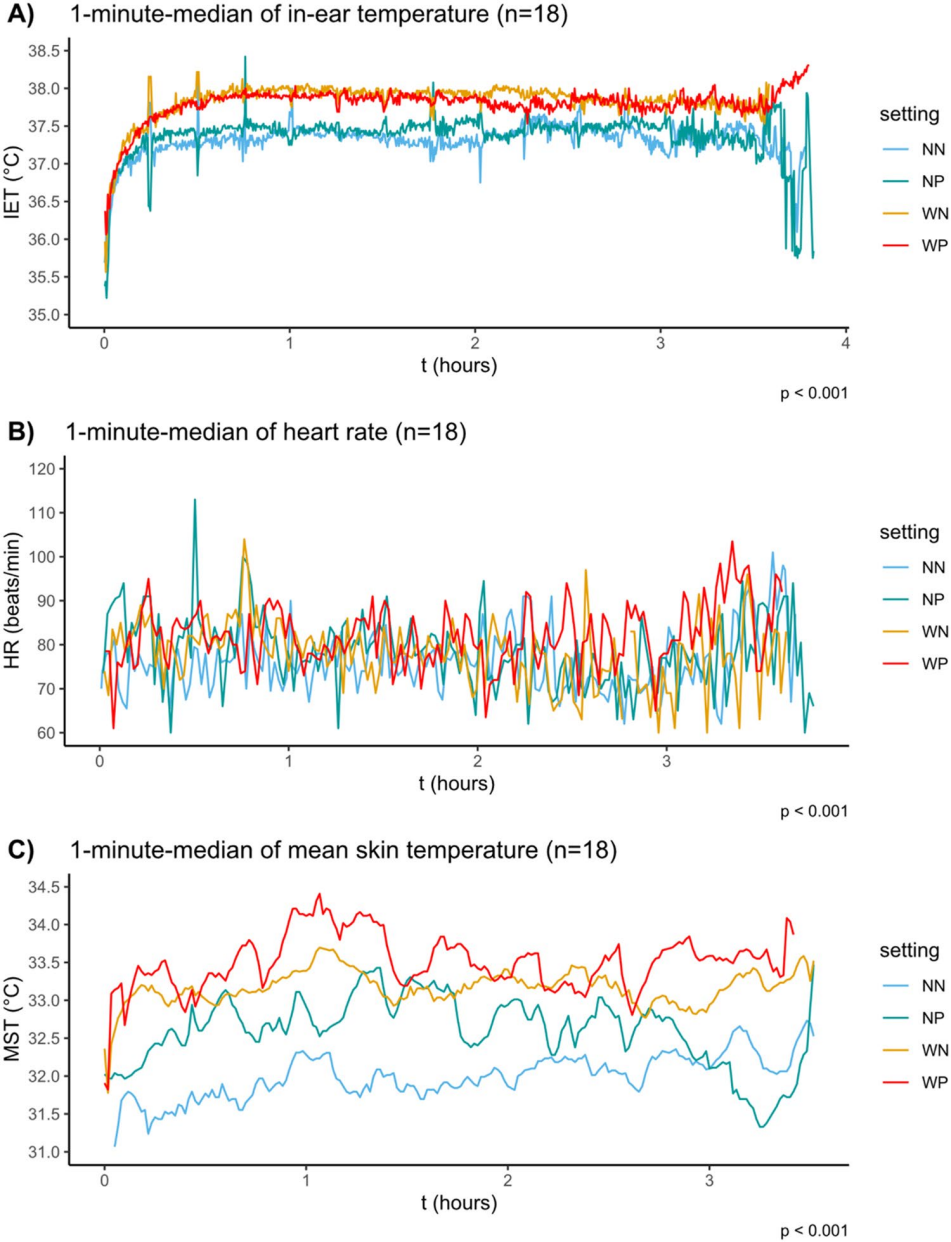
1 min median in-ear temperature (IET, **A**), heart rate (HR, **B**) and mean skin temperature (MST, **C**) calculated from the results of all participants. The applied settings were a) 22 °C (NN), b) 22 °C and PPE (NP), c) 27 °C (WN) and d) 27 °C and PPE (WP)

For trials under normal temperature (NN and NP), the average dry bulb temperature was 23.0 °C (heat index of 21.2 °C) and the value was 27.3 °C (heat index of 27.2 °C) for the trials under warm temperatures (WN and WP). The measured mean relative humidity for all settings was 34 ± 5.2%. The results of in IET, HR and MST during all experimental sessions are shown in Fig. 2: The data is presented as one-minute-medians calculated from the results of all participants (n = 18). Additionally, a non-time-resolved representation of the data as box-whisker-plots can be found in the supplemental information (Fig S2).

Concerning participants´ IET, the median values during experimental sessions under warm temperatures were higher than those under normal temperatures (37.3 °C, 37.4 °C, 37.8 °C, and 37.9 °C for NN, NP, WN and WP, respectively). Using the identical order, the median values for participants´ HR were 76, 78, 79, and 83 beats/min. The median values for participants´ MST were 32.0 °C, 32.4 °C, 33.0 °C, and 33.4 °C for NN, NP, WN and WP, respectively.

Using the ANOVA (i.e., Wilcoxon rank-sum test), the differences in each physiological parameter between all four trial settings were found as significant (*p* < 0.001). However, as this method is not robust against systematic interindividual variability, the data were re-analyzed and predicted using the linear mixed-model regression.

### Prediction model using linear mixed regression analysis

**Fig. 3:**
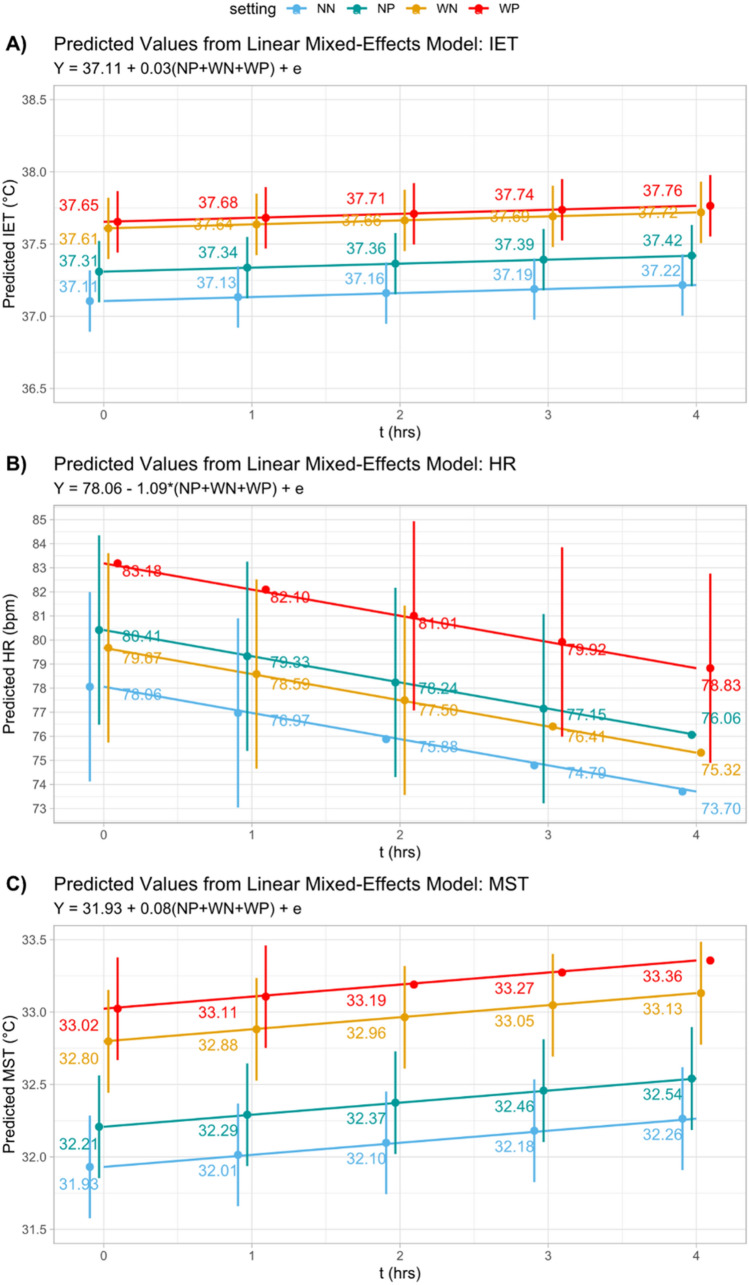
Linear mixed-effects models for in-ear temperature (IET, **A**), heart rate (HR, **B**) and mean skin temperature (MST,** C**). The applied settings were a) 22 °C (NN); b) 22 °C and PPE (NP); c) 27 °C (WN); and d) 27 °C and PPE (WP)

To analyze the interindividual differences in the physiological data, a mixed-model regression analysis was performed. This model takes within- and between-subjects variance into account. The term “mixed model” refers to the inclusion of both fixed effects, which are model components used to define systematic relationships such as the physiological changes over time and/or experimentally induced group differences, as well as random effects, which account for variability among participants around the systematic relationships captured by the fixed effects (Van Dongen et al. 2004; Ravindra et al. 2019). Figure 3 illustrates our regression model.

Despite individual variances, among 18 participants, compared to the initial session (NN), significant increments were found within all trials (*p* < 0.001), particularly during the session WP (+ 0.5 °C for IET; + 5 beats/min for HR and + 1.1 °C for MST). The regression coefficients of all measured parameters are listed in Table 2.

**Table 2:** Prediction of physiological parameters using a linear mixed-effects model (n = 18)

	In-ear temperature (°C)	Heart rate (beats/min)	Mean skin temperature (°C)
Constant (NN)	37.11*** (0.11)	78.06*** (2.01)	31.93*** (0.18)
Time	0.03*** (0.00)	− 1.09*** (0.02)	0.08*** (0.01)
NP	0.20*** (0.00)	2.36*** (0.06)	0.28*** (0.02)
WN	0.50*** (0.00)	1.62*** (0.06)	0.87*** (0.02)
WP	0.55*** (0.00)	5.12*** (0.06)	1.1*** (0.02)

Standard errors are given in parenthesis

The applied settings (NN: 22 °C, NP, 22 °C and personal protective equipment (PPE), WN: 27 °C and WP: 27 °C and PPE) were included as fixed effects in the regression model

***p < 0.001, **p < 0.01, *p < 0.05

## Discussion

In our study, we focused on HCW since there is a substantial research gap on heat strain for this occupation. We postulated that the heat strain among HCW generated from their physical activities is exacerbated by the heat stress due to increased indoor temperatures. This situation can be further aggravated due to the wearing of PPE, as it has been mandatory caring for patients with SARS-CoV-2 or other infectious diseases. Based on these hypotheses, our cross-over study revealed that the combination of internal and external heat stress while conducting HCW-related activities in the climate chamber induces a significant increase in all observed physiological parameters. This increment was particularly high when performing the experimental scenario wearing PPE at 27 °C. Such findings support previous studies concerning the adverse effects of occupational heat strain, particularly attributable to the wearing of PPE (Dorman and Havenith 2009; Eggenberger et al. 2018; Foster et al. 2020b).

The measurement of the IET as a proxy of the body temperature and HR using wearables was based on existing studies as it has been shown that such physiological parameters can provide indicators of health status and have tremendous diagnostic value (Eggenberger et al. 2018). Additionally, we realized that measuring participants´ MST is also important, as it is considered an indicator of thermal sensation. Although the thermoregulatory mechanisms of healthy humans allow only minor changes in core temperature, peripheral skin temperatures respond clearly to changes in ambient temperature or metabolism (Krishnamurthy et al. 2017).

Besides the increase in the observed physiological parameters (IET, HR and MST), participants frequently reported drinking more water, feeling tired, and sweating excessively during the experiments with PPE (Quartucci et al., Effects of heat and personal protective equipment on thermal strain in health care workers—Part A: Application of a standardized protocol and assessment of subjective well-being, submitted for publication).

It is well known that heat stress reduces the human capability to perform activities at full capacity due to the physiological dysfunction (Russo et al. 2019; Foster et al. 2020b). In the study by Gostimorovic et al. concerning heat stress on human cardiovascular functions, a vigorous, long-term impairment of physiological parameters did lead to several health issues such as heart attacks, malignant cardiac arrhythmias, thromboembolic diseases and heat-induced sepsis like shock. (Gostimirovic et al. 2020).

As climate change progresses, the frequency of hot days with uncomfortable indoor temperatures is expected to increase. This situation can lead to heat strain events when the core body temperature exceeds its normal level, resulting from a total heat load exceeding the capacity for heat dissipation. This may cause long-term health and productivity risks with devastating economic consequences (Russo et al. 2019).

Finally, the feasibility of using *Cosinuss° Two* in-ear sensor^®^ to observe the physiological response which is attributable as heat stress was investigated. While some studies described the advantages of this device (Burgos et al. 2020; Ellebrecht et al. 2022), some evaluated the inaccuracy of using this wearable, particularly in measuring core body temperature (Roossien et al. 2020, 2021). Consequently, we used the terminology of IET instead of core body temperature. Nevertheless, the IET is a good proxy of the core body temperature. Since the goal of our study is observing the physiological responses of HCW under different experimental conditions, this device is suitable as a monitoring tool for effects caused by thermal stress. Furthermore, the potential measurement error due to wind factor was likely minimized due to controlled experimental environment in the climate chamber.

## Strength and limitations

To our knowledge, this is the first experimental study concerning the heat stress measurement by conducting simulations of the HCW-related activities in a controlled climatic environment. Besides supporting existing knowledge, this study provides valuable information about planning heat stress experiments using realistic but controlled conditions. Furthermore, the feasibility of using wearables to assess heat stress was demonstrated. A limiting factor is the temperature measurement in the ear, which is a proxy of the core body temperature. However, we considered this more feasible and non-invasive compared to other methods for the determination of the core body temperature. Furthermore, data quality of the ear sensors was impaired in cases when the sensor was not correctly placed in the ear or slipped out. This happened more frequently when the participants had to take off FFP2 masks. In this case, the experiment was stopped for a brief moment and a member of the study team reinserted the sensor. At last, the temperature and humidity in the climate chamber were not as stable as intended. However, the temperature difference between the settings was significantly higher than its variance. For relative humidity, the variance likely did not have an influence on the results.

## Conclusions

In summary, our results suggest that the combination of internal heat stress triggered by high physical activity as well as external heat stress induced by increased environmental heat appears to be related to health and productivity losses of workers engaged in the healthcare sector. This situation can be worsened by wearing additional PPE, which was required in particular working conditions. This fact was supported by both participants’ perceptions and physiological measurements. All nations, particularly during a worldwide pandemic such as COVID-19, are dependent on HCW. Subsequently, their health and welfare are of paramount importance for sustained health stability. Unfortunately, HCW are likely at high risk of the health burden due to excessive occupational heat exposure. Lack of ventilation or non-air-conditioning building, as it is common in Germany, can aggravate this situation. As a consequence, adequate cooling provisions or other mitigation strategies should be implemented in order to reduce potential heat strain in HCW (Foster et al. 2020a; Lou et al. 2021; Bongers et al. 2022). Further research concerning the current and future risks of occupational heat exposure is crucial to develop comprehensive evidence-based policies for protecting HCW from the adversities of heat stress. We hope that the results of this study will help policymakers establishing appropriate interventions based on HCW’s work-related health hazards due to heat stress.

### Supplementary Information

Below is the link to the electronic supplementary material.

Supplementary file1 (PDF 94 KB)

Supplementary file2 (PDF 452 KB)

## Data Availability

The data can be made available upon reasonable request.
