# Supplementary material for: Effects of heat and personal protective equipment on thermal strain in healthcare workers: part B—application of wearable sensors to observe heat strain among healthcare workers under controlled conditions
Source: Int Arch Occup Environ Health. 2023 Nov 10;97(1):35–43. doi: 10.1007/s00420-023-02022-2 (PMC10791845; doi:10.1007/s00420-023-02022-2)
Supplement: Supplementary file 1 — Supplementary file1 (PDF 94 KB) [file 420_2023_2022_MOESM1_ESM.pdf]

Protocol for simulations with patients ID: _____

Date: _____

Patient 1

1. walking on a treadmill at 5 km/h (5 min) _____

2. simulation of measuring vital parameters (4 min) _____
    a. put on gloves
    b. put on additional PPE (optional according to experiment)
    c. apply blood pressure monitor to dummy
    d. simulate measurement
    e. remove blood pressure monitor from dummy

3. transfer dummy from bed to chair (3 min) _____

4. simulate intimate hygiene with dummy (3 min) _____
    a. washing
    b. change gloves

5. change linen (8 min) _____

6. transfer dummy from chair to bed (3 min) _____

7. simulate partial body hygiene (15 min) _____
    a. sit up dummy and wash back
    b. intimate hygiene
    c. change gloves
    d. wash legs and dry
    e. sit up and lie down dummy

8. clean up and disinfect working area (3 min) _____
    a. remove additional PPE (optional according to experiment)
    b. remove gloves

9. walking on a treadmill at 4 km/h (10 min) _____

10. sit down at desk and rest (15 min) _____

Patient 2

1. walking on a treadmill at 5 km/h (5 min) _____

2. simulation of measuring vital parameters (4 min) _____
    a. put on gloves
    b. put on additional PPE (optional according to experiment)
    c. apply blood pressure monitor to dummy
    d. simulate measurement
    e. remove blood pressure monitor from dummy

3. transfer dummy from bed to chair (3 min) _____

4. simulate intimate hygiene with dummy (3 min) _____
    a. washing
    b. change gloves

5. change linen (8 min) _____

6. transfer dummy from chair to bed (3 min) _____

7. simulate full body hygiene (15 min) _____
    a. undress dummy
    b. wash dummy head to toe
    c. change gloves
    d. intimate hygiene
    e. change gloves
    f. dry body
    g. simulate skin care
    h. dress dummy

8. clean up and disinfect working area (3 min) _____
    a. remove additional PPE (optional according to experiment)
    b. remove gloves

9. walking on a treadmill at 4 km/h (10 min) _____

10. sit down at desk and rest (15 min) _____

Patient 3

1. walking on a treadmill at 5.8 km/h (5 min) _____

2. simulation of measuring vital parameters (4 min) _____
    a. put on gloves
    b. put on additional PPE (optional according to experiment)
    c. apply blood pressure monitor to dummy
    d. simulate measurement
    e. remove blood pressure monitor from dummy

3. transfer dummy from bed to chair (3 min) _____

4. simulate intimate hygiene with dummy (3 min) _____
    a. washing
    b. change gloves

5. change linen (8 min) _____

6. transfer dummy from chair to bed (3 min) _____

7. simulate full body hygiene (15 min) _____
    a. undress dummy
    b. wash dummy head to toe
    c. change gloves
    d. intimate hygiene
    e. change gloves
    f. dry body
    g. simulate skin care
    h. dress dummy

8. clean up and disinfect working area (3 min) _____
    a. remove additional PPE (optional according to experiment)
    b. remove gloves

9. walking on a treadmill at 4 km/h (10 min) _____

10. sit down at desk and rest (15 min) _____
