# Supplementary material for: Effects of heat and personal protective equipment on thermal strain in healthcare workers: part B—application of wearable sensors to observe heat strain among healthcare workers under controlled conditions
Source: Int Arch Occup Environ Health. 2023 Nov 10;97(1):35–43. doi: 10.1007/s00420-023-02022-2 (PMC10791845; doi:10.1007/s00420-023-02022-2)

Effects of heat and personal protective equipment on thermal strain in healthcare workers - Part B: Application of wearable sensors to observe heat strain among healthcare workers under controlled conditions *

Supplemental information

Figure S1: Pictures of the climate chamber setup

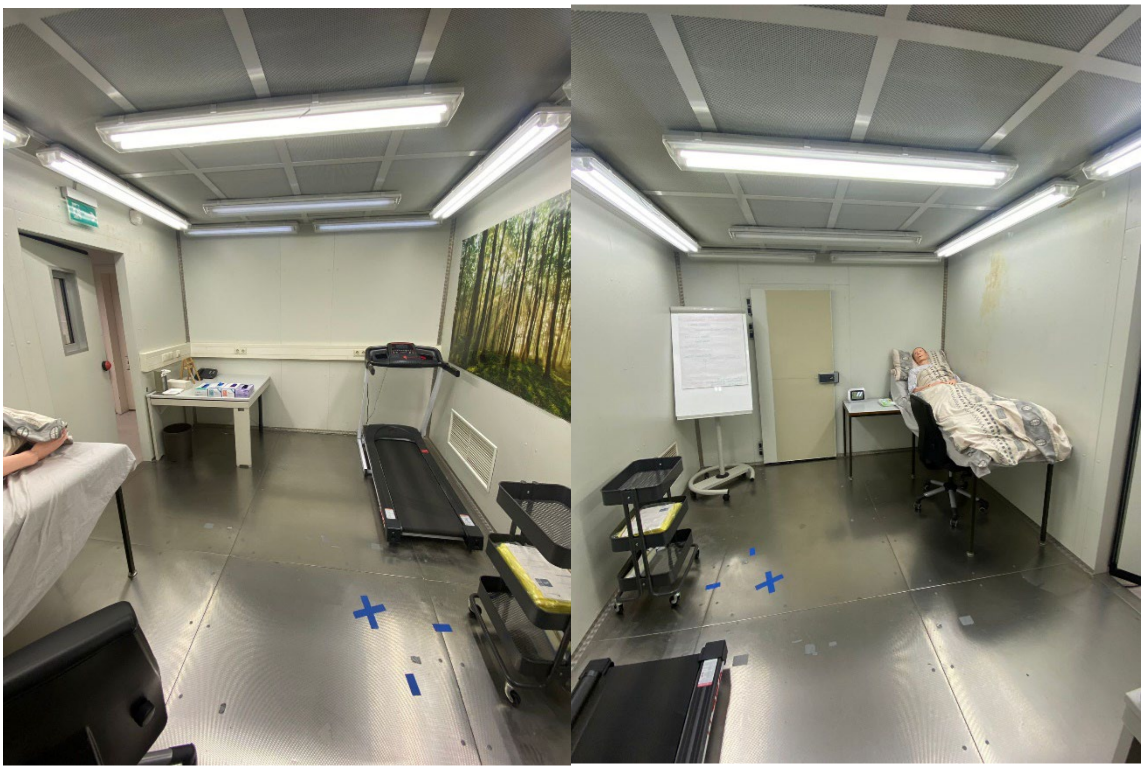

Figure S2: Boxplots of all measured physiological parameters: in-ear temperature (A), heart rate (B) and mean skin temperature (C). The applied settings were a) 22 °C (NN), b) 22 °C and PPE (NP), c) 27 °C (WN) and d) 27 °C and PPE (WP).

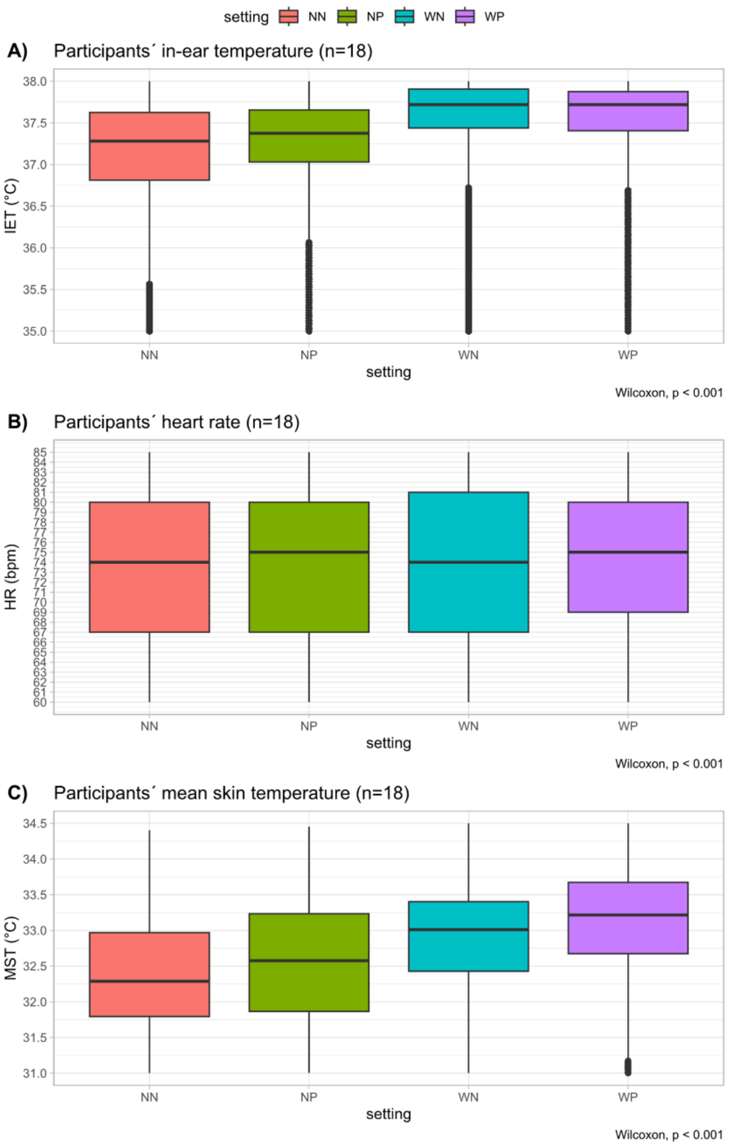

Supplement: Supplementary file 2 — Supplementary file2 (PDF 452 KB) [file 420_2023_2022_MOESM2_ESM.pdf]
